# Selection of two pronuclei zygotes before electroporation does not improve gene editing efficiency or reduce mosaicism in porcine embryos despite optimized timing and Hoechst staining

**DOI:** 10.14202/vetworld.2026.2406-2417

**Published:** 2026-06-13

**Authors:** Koki Takebayashi, Takeshige Otoi, Manita Wittayarat, Oky Setyo Widodo, Theerawat Tharasanit, Kaywalee Chatdarong, Yuichiro Nakayama, Zhao Namula, Megumi Nagahara, Maki Hirata, Fuminori Tanihara

**Affiliations:** 1 Bio-Innovation Research Center, Tokushima University, Tokushima 779-3233, Japan; 2 Faculty of Veterinary Medicine, Universitas Airlangga, Surabaya 60115, Indonesia; 3 Faculty of Veterinary Science, Chulalongkorn University, Bangkok 10330, Thailand; 4 College of Coastal Agricultural Sciences, Guangdong Ocean University, Zhanjiang 524088, China; 5 Faculty of Veterinary Science, Prince of Songkla University, Songkhla 90110, Thailand

**Keywords:** blastocyst development, CRISPR/Cas9, electroporation, genome editing, Hoechst 33342, mosaicism, porcine embryos, two pronuclei

## Abstract

**Background and Aim:**

Electroporation-mediated delivery of Clustered-Regularly Interspaced Short Palindromic Repeats (CRISPR)/CRISPR-associated protein 9 components is an effective approach for genome editing in porcine embryos; however, mosaicism remains a major limitation. Selecting normally fertilized zygotes with two pronuclei (2PN) before electroporation may improve editing uniformity and reduce mosaicism. Therefore, this study investigated the effects of 2PN selection, optimized timing of pronuclear observation, and Hoechst 33342 staining conditions on embryo development and genome editing efficiency in porcine embryos.

**Materials and Methods:**

In the first experiment, pronuclear dynamics after *in vitro* fertilization (IVF) were evaluated by collecting zygotes at 8–24 h post-fertilization, followed by centrifugation, fixation, and Hoechst 33342 staining. In the second experiment, the effects of Hoechst 33342 concentration (5 and 10 μg/mL) and exposure duration (10 and 30 min) on embryo development were assessed using live zygotes collected 20 h after fertilization. In the third experiment, zygotes with visible 2PN selected after centrifugation and Hoechst staining were electroporated with guide RNA targeting the *TP53* gene. Developmental competence, mutation efficiency, and mosaicism were compared with non-selected control zygotes.

**Results:**

The proportion of zygotes with 2PN gradually increased after fertilization and reached the highest level (47.4%) at 20 h post-IVF before significantly declining at 24 h (p < 0.05). Among the staining conditions tested, treatment with 5 μg/mL Hoechst 33342 for 30 min resulted in the highest blastocyst formation rate (14.3%, p < 0.05). Following electroporation, blastocyst development rates did not differ significantly between the 2PN-selected group (6.5%) and the control group (10.8%). Similarly, the biallelic mutation rate and total mutation efficiency were comparable between selected zygotes (78.6% and 85.5%, respectively) and controls (81.8% and 92.5%, respectively).

**Conclusion:**

Selection of porcine zygotes with 2PN before electroporation did not improve genome editing efficiency or reduce mosaicism despite optimization of pronuclear observation timing and Hoechst staining conditions. The persistence of mosaicism may be associated with asynchronous DNA replication between maternal and paternal pronuclei. Although 2PN selection was safe for embryo development, additional strategies targeting pronuclear synchronization and replication timing are required to achieve more uniform genome editing in porcine embryos.

## INTRODUCTION

Electroporation serves as an alternative approach for delivering Clustered-Regularly Interspaced Short Palindromic Repeats (CRISPR)–associated protein 9 (CRISPR/Cas9) genome editing components into a variety of zygotes [1–3]. However, similar to other genome editing techniques, electroporation frequently results in mosaicism, in which cells with different genotypes coexist within the same animal [1, 4–6]. Introducing editing reagents at or shortly after fertilization may reduce mosaicism [1]. In mice, delivery of Cas9/sgRNA ribonucleoprotein (RNP) complexes into early-stage zygotes through electroporation, rather than microinjection, successfully eliminated mosaic mutants, suggesting that genome editing occurred before the first DNA replication event [7]. However, electroporation is generally performed without confirming the pronuclear status of embryos, meaning that some zygotes may possess only one pronucleus or fail to form pronuclei altogether. This lack of verification may increase mosaicism and reduce genome editing efficiency. Electroporation-mediated genome editing in porcine embryos has been reported to achieve high editing efficiencies, often exceeding 80%, depending on the experimental conditions [8, 9]. Therefore, selecting properly fertilized zygotes with two pronuclei (2PN) may further improve editing outcomes. Nevertheless, DNA replication in mammalian zygotes is not always synchronized between maternal and paternal pronuclei. Previous studies in mice demonstrated that DNA synthesis begins earlier in the female pronucleus, whereas the male pronucleus replicates later, resulting in asynchronous S-phase progression [10, 11]. Such asynchrony may cause differences in the timing of CRISPR/Cas9-mediated genome editing and contribute to persistent mosaicism, even in normally fertilized zygotes with 2PN. To address this issue, we focused on selecting embryos with 2PN at a defined time after *in vitro* fertilization (IVF) to improve genome editing outcomes following electroporation.

When selecting porcine embryos with 2PN, several critical factors must be considered, including the timing of zygote collection after fertilization and the parameters of DNA staining, such as concentration and exposure duration. *In vitro* fertilized porcine zygotes exhibit male pronucleus formation between 6 and 12 h post-insemination [12]. Furthermore, both male and female pronuclei have been reported to develop in a relatively synchronized manner between 16 and 32 h post-insemination [12]. This synchronization is important for normal embryonic development because coordinated pronuclear progression supports appropriate genome activation and cell division. However, the broad time window for 2PN formation, which may vary among laboratory conditions and IVF systems, necessitates the identification of an optimal collection time to maximize the proportion of zygotes with visible 2PN. Another important consideration is the optimization of DNA staining for pronuclear visualization. In livestock species such as pigs, goats, and cattle, oocytes and embryos contain abundant intracellular lipid droplets that obscure nuclear structures and complicate the identification of pronuclear stages [13]. In this context, Hoechst 33342 serves as a useful fluorescent DNA stain for evaluating nuclear stages in living oocytes and embryos [13]. However, Hoechst staining may negatively affect embryo quality and developmental competence. In goat oocytes, Hoechst 33342 reduced both total and normal fertilization rates, particularly when applied during the early stages of *in vitro* maturation [13]. Similarly, in parthenogenetically activated mouse oocytes, blastocyst formation rates were significantly reduced because of the combined toxicity of Hoechst staining and UV irradiation, despite normal activation and cleavage rates [14]. Although Hoechst staining has been used for pronuclear visualization and polyspermy assessment in various species, systematic optimization of Hoechst concentration and exposure duration to minimize developmental toxicity during CRISPR/Cas9 electroporation in porcine embryos remains insufficiently investigated. Therefore, further studies are required to establish safe and effective staining conditions that enable reliable pronuclear visualization while preserving embryonic viability.

Despite the increasing application of electroporation-mediated CRISPR/Cas9 genome editing in porcine embryos, mosaicism remains a major obstacle limiting the production of uniformly edited animals. Previous studies primarily focused on optimizing electroporation parameters, guide RNA design, and developmental stages for reagent delivery, whereas limited attention has been given to the biological status of the zygote before electroporation [8, 9]. In particular, most porcine electroporation studies have applied genome editing reagents without verifying pronuclear formation or synchrony, even though asynchronous DNA replication between maternal and paternal pronuclei has been recognized as a potential contributor to mosaicism [10, 11]. Furthermore, although pronuclear visualization using Hoechst staining has been reported in mammalian embryos, there is a lack of systematic studies evaluating the optimal timing of 2PN observation, safe Hoechst staining conditions for live porcine embryos, and the combined effects of these procedures on subsequent genome editing efficiency and embryonic development. Therefore, the practical value of selecting zygotes with 2PN before electroporation, particularly under optimized staining and timing conditions, remains unclear in porcine embryos.

Therefore, this study aimed to investigate whether selecting porcine zygotes with 2PN before electroporation could improve CRISPR/Cas9-mediated genome editing efficiency and reduce mosaicism. Specifically, this study evaluated the temporal dynamics of pronuclear formation following IVF to determine the optimal timing for 2PN selection, assessed the effects of different Hoechst 33342 concentrations and exposure durations on embryo development, and examined the developmental competence, mutation efficiency, and mosaicism rates of embryos electroporated after 2PN selection. In addition, this study sought to establish a practical and optimized workflow integrating pronuclear visualization, live embryo selection, and electroporation for porcine genome editing applications.

## MATERIALS AND METHODS

All procedures were performed at 39°C unless otherwise stated, and all media were equilibrated for 2 h before use.

### Ethical approval

All animal-related procedures were reviewed and approved by the Institutional Animal Care and Use Committee of Tokushima University, Tokushima, Japan (approval number: T2025-6; approved on April 9, 2025). All experimental procedures were conducted in accordance with institutional guidelines and regulations for the ethical use and care of animals in research. Pig ovaries used in this study were collected from animals slaughtered for commercial food production, and no animals were euthanized specifically for this research purpose.

### Study period and location

The study was conducted from September to December 2025 at the Bio-Innovation Research Center, Tokushima University, Tokushima, Japan. Oocyte collection, *in vitro* embryo production, electroporation, and molecular analyses were performed in the laboratory facilities of the center under controlled environmental conditions.

### Study design

This study was designed to investigate whether selecting porcine zygotes with two pronuclei (2PN) before electroporation could improve CRISPR/Cas9-mediated genome editing efficiency and reduce mosaicism. The experimental design consisted of three sequential experiments. Experiment 1 evaluated the temporal dynamics of pronuclear formation after IVF to determine the optimal timing for 2PN observation. Experiment 2 assessed the effects of different Hoechst 33342 concentrations and exposure durations on embryo development following pronuclear visualization. Experiment 3 examined the developmental competence, mutation efficiency, and mosaicism of embryos electroporated after 2PN selection. Zygotes were randomly allocated to treatment groups in each replicate to minimize experimental bias.

### Oocyte collection and *in vitro* maturation

Oocyte collection, *in vitro* maturation, fertilization, and embryo culture were performed as previously described [15]. Briefly, pig ovaries were obtained from prepubertal crossbred gilts (Landrace × Large White × Duroc) at a local slaughterhouse. The donor animals were approximately 6 months of age, weighed approximately 110 kg, and were clinically healthy. Cumulus-oocyte complexes (COCs) with a uniform ooplasm and compact multilayered cumulus cell mass, indicative of normal oocyte morphology, were selected and cultured in maturation medium at 39°C in a humidified incubator containing 5% CO_2_.

The maturation medium consisted of 25 mM HEPES tissue culture medium 199 with Earle’s salts (TCM 199; Invitrogen Co., Carlsbad, CA, USA; Cat. No. 11150-059) supplemented with 10% (v/v) porcine follicular fluid, 0.6 mM cysteine (Sigma-Aldrich, St. Louis, MO, USA), 50 µM sodium pyruvate (Sigma-Aldrich), 2 mg/mL D-sorbitol (Fujifilm Wako Pure Chemical Co., Osaka, Japan), 50 µM β-mercaptoethanol (Fujifilm Wako Pure Chemical Co.; Cat. No. M3148), 1 µg/mL 17 β-estradiol (Sigma-Aldrich; Cat. No. E8875), 10 IU/mL equine chorionic gonadotropin (Celarmon 1000; Kyoritu Seiyaku, Tokyo, Japan), 10 IU/mL human chorionic gonadotropin (Gestron; Kyoritu Seiyaku), and 50 µg/mL gentamicin (Sigma-Aldrich; Cat. No. G3632). After 20–22 h of maturation, the COCs were cultured for an additional 24 h in maturation medium without hormones under the same atmospheric conditions.

### IVF

For IVF, frozen-thawed spermatozoa were transferred to 6 mL porcine fertilization medium (PFM; Research Institute for the Functional Peptides Co., Yamagata, Japan; Cat. No. IFP1020P), which was prepared according to the formulation described by Yoshioka *et al*. [16], and washed by centrifugation at 500 × g for 5 min. After centrifugation, pelleted spermatozoa were resuspended in PFM and adjusted to a final concentration of 5 × 10^6^ cells/mL. The matured oocytes were transferred into four-well dishes containing 500 µL PFM supplemented with frozen-thawed spermatozoa per well and covered with mineral oil. The gametes were co-incubated for 5 h at 39°C under 5% CO_2_, 5% O_2_, and 90% N_2_. Sperm samples were evaluated before use, and only samples with stable fertilization performance were selected to minimize polyspermy.

### Embryo culture

After IVF, putative zygotes were denuded from cumulus cells and attached spermatozoa by mechanical pipetting and transferred to porcine zygote medium (PZM-5; Research Institute for the Functional Peptides Co.; Cat. No. IFP0410P), originally described by Yoshioka *et al*. [16]. Zygotes were cultured continuously *in vitro* at 39°C in a humidified incubator containing 5% CO_2_, 5% O_2_, and 90% N_2_. Under standard IVF conditions, the fertilization rate at 10 h after insemination was approximately 40%, and approximately 70% of fertilized oocytes exhibited normal fertilization with 2PN [17].

All cleaved embryos were transferred into 100 µL droplets of porcine blastocyst medium (PBM; Research Institute for the Functional Peptides Co.; Cat. No. IFP1030P), a modified version of PZM optimized for blastocyst development, at 72 h after insemination. Embryos were subsequently cultured for an additional 4 d to evaluate blastocyst development and genome editing efficiency.

### Centrifugation treatment

Putative zygotes were collected at different time points after IVF initiation and centrifuged to polarize intracellular lipid droplets because our previous study suggested that lipid droplet polarization by high-speed centrifugation could influence electroporation efficiency without adversely affecting embryo development [18]. Zygotes were transferred into 2 mL Sarstedt screw-cap tubes containing 500 µL PZM-5 and centrifuged at 10,000 × *g* for 10 min at 35.0 ± 1.0°C using a high-speed refrigerated centrifuge (MX-307; Tomy Seiko, Tokyo, Japan) to polarize intracellular lipid droplets [19].

### Electroporation

Electroporation was performed after centrifugation as previously described [20]. Briefly, an electrode (LF501PT1-20; BEX, Tokyo, Japan) connected to a CUY21EDIT II electroporator (BEX) was placed under a stereoscopic microscope. Approximately 30–40 zygotes were washed with Opti-MEM I solution (Invitrogen Co.; Cat. No. 31985-062) and aligned within a 1-mm electrode gap in a chamber slide filled with 10 µL Opti-MEM I solution containing 200 ng/µL Cas9 protein (Guide-it™ Recombinant Cas9; Takara Bio, Shiga, Japan) and 100 ng/µL gRNA (Alt-R CRISPR-Cas9 crRNAs and Alt-R tracrRNA; Integrated DNA Technologies, IA, USA) targeting exon 3 of *TP53* (5´-GGTCTTCTGAGAAGGGACAA-3´), selected according to a previous study validating its genome editing efficiency [21].

The zygotes were electroporated at 25 V/mm for 1 msec with five pulses. After electroporation, embryos were washed three times in fresh culture medium and immediately cultured as described above.

### Genomic DNA extraction and polymerase chain reaction (PCR)

Genomic DNA was isolated from blastocysts by boiling in 10 µL 50 mM NaOH for 10 min at 98°C. After neutralization with 1 µL 1 M Tris-HCl (pH 8.0), genomic regions flanking the sgRNA target sequence were amplified by PCR using specific primers (5´-CGAACTGGCTGGATGAAAAT-3´ [forward] and 5´-CCAGGGTCCAAGGTCATAGA-3´ [reverse]) and KOD One DNA polymerase (TOYOBO, Osaka, Japan). PCR amplification was performed under the following conditions: initial denaturation at 98°C for 10 sec, followed by 40 cycles of annealing at 65°C for 5 sec and extension at 68°C for 15 sec. PCR products were purified by agarose gel electrophoresis using a FastGene Gel/PCR Extraction Kit (Nippon Genetics, Tokyo, Japan).

### Sequencing and genotyping

Targeted genomic regions of the PCR products were directly sequenced by Sanger sequencing using the BigDye Terminator Cycle Sequencing Kit version 3.1 (Thermo Fisher Scientific, Waltham, MA, USA) and an ABI 3500 Genetic Analyzer (Applied Biosystems, Foster City, CA, USA). Sequencing was performed on all blastocysts; however, only blastocysts with successful sequencing results were included in subsequent analyses (Table 2).

**Table 1:** Effects of Hoechst 33342 concentration and exposure time after centrifugation on the development of zygotes with two pronuclei*.

Concentration of Hoechst 33342 (μg/mL)**	Exposure time (min)**	No. of zygotes examined	No. of zygotes cultured (n/N) (%)***	Cleaved embryos (n/N) (%)	Embryos developed to blastocysts (n/N) (%)
5	10	262	103/262 (39.0 ± 4.1)	89/103 (85.0 ± 2.8)	7/103 (5.7 ± 1.8)^a^
5	30	264	97/264 (37.2 ± 1.9)	84/97 (83.8 ± 4.7)	12/97 (14.3 ± 3.1)^b^
10	10	261	110/261 (42.3 ± 2.2)	88/110 (71.9 ± 11.3)	7/110 (5.3 ± 1.4)^a^
10	30	261	115/261 (44.4 ± 2.9)	94/115 (80.3 ± 3.2)	10/115 (6.7 ± 1.8)^a^

* Percentages are expressed as mean ± SEM. Values are presented as percentages with absolute numbers (n/N). Data were obtained from five independent replicates.

** Zygotes were centrifuged at 10,000 × g for 10 min at 20 h after the start of *in vitro* fertilization. After centrifugation and staining with Hoechst 33342, only zygotes with two visible pronuclei were cultured for 7 d.

*** Only zygotes with two pronuclei were cultured for 7 d.

^a,b^ Values with different superscript letters in the same column are significantly different (p < 0.02).

**Table 2:** Development and mutation of zygotes with two pronuclei after selection by centrifugation and staining with Hoechst 33342*.

Selection of zygotes with two pronuclei**	No. of zygotes examined	Cleaved embryos (n/N) (%)	Embryos developed to blastocysts (n/N) (%)	No. of blastocysts examined***	Biallelic mutation (n/N) (%)****	Mosaic mutation (n/N) (%)****	Wild-type (n/N) (%)****	Total mutation efficiency (mean ± SEM)
−	250	206/250 (82.4 ± 2.8)	27/250 (10.8 ± 3.0)	22	18/22 (81.8)	2/22 (9.1)	2/22 (9.1)	92.5 ± 4.5
+	247	193/247 (78.1 ± 1.3)	16/247 (6.5 ± 0.5)	14	11/14 (78.6)	3/14 (21.4)	0/14 (0.0)	85.5 ± 7.3

* Percentages are expressed as mean ± SEM. Values are presented as percentages with absolute numbers (n/N). Data were obtained from five independent replicates.

** Zygotes were centrifuged at 10,000 × *g* for 10 min at 20 h after the start of *in vitro* fertilization. After centrifugation, zygotes with (+) and without (−) selection by staining with 5 μg/mL Hoechst 33342 for 30 min were cultured for 7 d after electroporation with gRNAs targeting *TP53*.

*** Sequencing was performed on all blastocysts, the numbers shown represent the blastocysts that were successfully sequenced and included in the analysis.

**** Percentages were calculated by dividing the number of gene-edited blastocysts by the number of examined blastocysts.

The TIDE (Tracking of Indels by DEcomposition) web tool (https://tide.nki.nl) was used to analyze Sanger sequencing data. Sequence quality was evaluated based on chromatogram signal clarity, and only sequences with consistently clear peak resolution across the entire trace were included in the analysis. Sequence traces were aligned to wild-type reference sequences using default alignment parameters. The decomposition window was set at 10–50 bp downstream of the predicted Cas9 cleavage site, and indels within ±30 bp were analyzed. The quality of decomposition was assessed using the coefficient of determination (R²), and only results with R² ≥ 0.9 were considered for further analysis.

**Figure 1:**
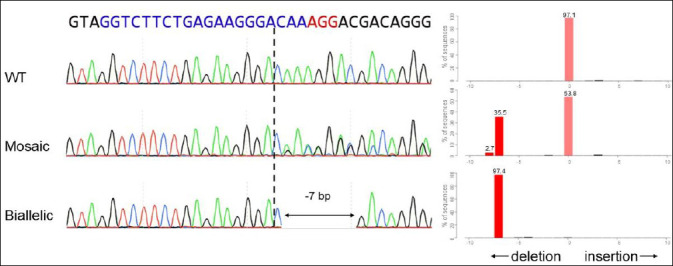
Representative results of Sanger sequencing of blastocysts after genome editing with guide RNA (gRNA) targeting *TP53* and frequencies of indels decomposed from Sanger sequencing data using tracking of indels by decomposition (TIDE) analysis. (WT) Analysis of wild-type blastocysts. (Mosaic) Analysis of blastocysts carrying mosaic mutations. The Sanger sequencing traces of mosaic blastocysts consisted of composite sequence traces after the cleavage site. (Biallelic) Analysis of a blastocyst carrying a biallelic mutation. The Sanger sequencing trace consisted of a 7-bp deletion without a wild-type sequence. Nucleotides in blue indicate target sequences, and nucleotides in red indicate protospacer adjacent motif sequences. Dashed lines indicate predicted cleavage sites.

Blastocysts were classified as biallelic mutants when no wild-type sequence signal was detected (wild-type frequency <5%) (Figure 1). Blastocysts were classified as mosaic mutants when multiple indel sequences were detected together with residual wild-type sequences (wild-type frequency ≥5%). Blastocysts were classified as wild-type when only wild-type sequences were detected without indel signals. Editing rate was defined as the ratio of gene-edited blastocysts to the total number of sequenced blastocysts. Editing efficiency was defined as the proportion of indel mutation events among blastocysts carrying mosaic or biallelic mutations. Off-target analyses were not performed in this study.

### Experimental design

In Experiment 1, the proportion of zygotes with 2PN was examined at different time points after IVF initiation. Zygotes were collected at 8, 10, 12, 15, 20, and 24 h after IVF initiation and centrifuged at 10,000 × *g* for 10 min as described above. After centrifugation, zygotes were fixed in 4% paraformaldehyde (Fujifilm Wako Pure Chemical Corp., Osaka, Japan) at 4°C overnight and permeabilized with 0.3% (w/v) polyvinylpyrrolidone and 1% (v/v) Triton X-100 (Sigma-Aldrich; Cat. No. T8787) for 15 min. Zygotes were then stained with 1.9 µM bisbenzimide (Hoechst 33342; Sigma-Aldrich; Cat. No. B2261; approximately 1.07 µg/mL) as previously described [22]. The number of zygotes with 2PN was examined using an epifluorescence microscope (Eclipse 80i; Nikon, Tokyo, Japan) equipped with a 355-nm excitation filter and a 420-nm barrier filter. Exposure time was limited to <1 sec per zygote to minimize phototoxicity. Different Hoechst staining conditions were used for fixed and live zygotes. In Experiment 1, fixed zygotes were stained, whereas in Experiments 2 and 3, live zygotes were stained before pronuclear selection.

**Figure 2:**
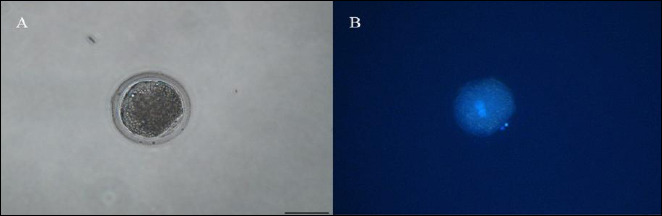
Representative images of porcine zygotes after centrifugation treatment. (A) Zygote before Hoechst 33342 staining. (B) Zygote with two visible pronuclei after Hoechst 33342 staining. Scale bar = 60 µm. The images are representative, and the proportion of zygotes showing clearly visible 2PN after staining was not quantitatively assessed in this study.

In Experiment 2, the effects of Hoechst 33342 concentration and exposure duration on embryo development were examined after centrifugation. Zygotes were collected at 20 h after IVF initiation because the proportion of zygotes with 2PN was highest at this time point in Experiment 1. Centrifuged zygotes were incubated in phosphate-buffered saline containing 5 or 10 µg/mL Hoechst 33342 and 0.3% polyvinylpyrrolidone for 10 or 30 min to compare staining conditions for pronuclear visualization (Figure 2). After staining, zygotes were transferred to 10 µL droplets of culture medium, and only zygotes with visible 2PN were selected using minimal exposure (<1 sec) to low-light filtered fluorescence. Selected zygotes were washed three times in fresh culture medium to remove residual Hoechst 33342 and cultured for 7 d as described above. However, the proportion of degenerated zygotes before culture was not quantitatively recorded.

In Experiment 3, embryo development and mutation efficiency were examined after selection of zygotes with 2PN by centrifugation and Hoechst 33342 staining. All zygotes were centrifuged at 10,000 × *g* for 10 min at 20 h after IVF initiation and stained with 5 µg/mL Hoechst 33342 for 30 min. Because blastocyst development was highest under this condition in Experiment 2, only zygotes with visible 2PN were electroporated with gRNA targeting *TP53* and cultured for 7 d. In the control group, all zygotes underwent identical centrifugation and staining procedures but were electroporated without pronuclear selection.

This study used an integrated workflow combining high-speed centrifugation for lipid droplet polarization, optimized low-concentration Hoechst 33342 staining (5 µg/mL for 30 min at 39°C) for live pronuclear visualization, and immediate electroporation of verified zygotes with 2PN after imaging. This workflow enabled the selection of properly fertilized embryos while minimizing toxicity and maintaining high-throughput processing. This methodological combination is novel in porcine CRISPR/Cas9-mediated genome editing studies. Additional refinements included minimal UV exposure (<1 sec per zygote) during fluorescence observation to reduce phototoxicity. These optimizations enabled reliable pronuclear identification in lipid-rich porcine zygotes and distinguished this protocol from conventional approaches based on fixed timing or non-selective staining.

Experiment 1 was performed in four independent replicates, whereas Experiments 2 and 3 were performed in five independent replicates (Supplementary Figure 1). Zygotes obtained from each IVF run were randomly allocated to treatment groups, and the total numbers are presented in Tables 1 and 2. Allocation procedures were performed to ensure comparable group sizes within each replicate.

### Statistical analysis

Percentage data for zygotes with 2PN and embryo development were arcsine-transformed before analysis to stabilize variance and analyzed by analysis of variance using the general linear model procedure in SAS for Windows version 9.1 (SAS Institute, Cary, NC, USA). When significant effects were detected, multiple comparisons were performed using Fisher’s protected least significant difference test.

In Experiment 2, the statistical model included Hoechst 33342 concentration, exposure duration, and their two-way interaction. If interactions were not significant, they were excluded from the model while retaining the main effects for analysis. In Experiment 3, embryo development and mutation efficiency data were analyzed using an independent Student’s t-test. The percentage of mutant blastocysts was analyzed using the chi-square test with Yates correction. Differences were considered statistically significant at p ≤ 0.05.

## RESULTS

### Pronuclear dynamics after IVF

**Figure 3:**
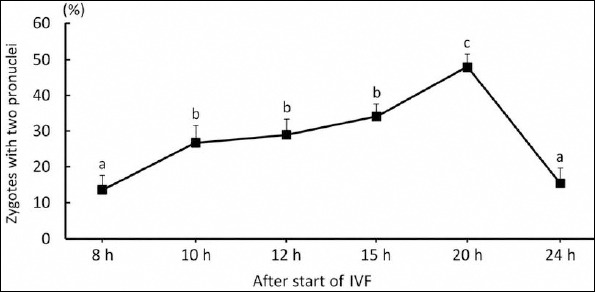
Dynamics of the ratio of porcine zygotes with two pronuclei after *in vitro* fertilization (IVF). Zygotes were centrifuged at 10,000 × *g* for 10 min at each time point (78–102 zygotes per group) after the start of IVF. After centrifugation, the number of zygotes with two pronuclei was examined by staining with Hoechst 33342. Four replicated trials were carried out. Bars represent the mean ± SEM. Values with different superscript letters are significantly different (p < 0.05).

When the dynamics of zygotes with 2PN after IVF were examined, the percentage of zygotes with 2PN gradually increased and reached the highest value at 20 h after the start of IVF (47.4%) (Figure 3). However, the percentage of zygotes with 2PN significantly decreased 4 h later, at 24 h after the start of IVF (p < 0.001). These findings provide a detailed temporal profile of pronuclear dynamics specific to this IVF system and may serve as a useful reference for optimizing zygote selection timing in future studies on porcine embryos.

### Effects of Hoechst 33342 concentration and exposure time on embryo development

Next, the effects of Hoechst 33342 concentration and exposure time after centrifugation on the development of zygotes with two visible pronuclei were evaluated (Table 1). No significant concentration × exposure time interaction was observed for the percentages of zygotes that cleaved or developed into blastocysts. There were no significant differences in cleavage rates among the treatment groups. However, the percentage of blastocyst formation in zygotes with 2PN confirmed by staining with 5 μg/mL Hoechst 33342 for 30 min was significantly higher than that in the other treatment groups (p < 0.02). This finding indicates that staining with 5 μg/mL Hoechst 33342 for 30 min represents an effective and practically useful condition for live pronuclear selection in porcine embryos.

### Effects of 2PN selection on embryo development and mutation efficiency

When the effects of selection treatment on the development and mutation of zygotes with two visible pronuclei after electroporation with gRNA targeting the *TP53* gene were examined, 2PN selection did not affect embryo development and did not improve the biallelic mutation rate or mutation efficiency of the resulting blastocysts (Table 2). The biallelic mutation rate was 78.6% in the 2PN-selected group and 81.8% in the non-selected control group. Similarly, total mutation efficiency was 85.5% in the 2PN-selected group and 92.5% in the non-selected control group. This finding indicates that verifying normal fertilization solely by 2PN selection is insufficient to improve genome editing outcomes or reduce mosaicism under the present experimental conditions.

## DISCUSSION

### Pronuclear dynamics and optimization of 2PN selection timing

Selecting porcine embryos with 2PN, particularly at the optimal post-fertilization timing, is important for improving genome editing outcomes. In the present study, we first determined the time point after fertilization that yielded the highest proportion of embryos with 2PN. Our findings demonstrated that the percentage of zygotes with 2PN gradually increased and reached a peak of 47.4% at 20 h after the start of IVF, but significantly declined within the following 4 h. These findings are consistent with a previous report showing a similar temporal trend in pronuclear visibility, in which pronuclear formation increased until 20 h post-IVF and decreased by 24 h, although that study did not quantify the number of pronuclei observed at each time point [23]. Synchronous pronuclear formation has been associated with normal fertilization and improved embryonic development in porcine embryos [24]. Therefore, precise timing at 20 h after IVF appears essential for maximizing the recovery of zygotes with 2PN because delayed collection may reduce the availability of appropriately fertilized embryos for subsequent experimental procedures.

### Effects of Hoechst 33342 staining on embryo development

Hoechst 33342 is widely used to visualize chromatin organization in live mammalian oocytes and zygotes and facilitates the identification of pronuclear formation and developmental stages [13]. Although it is considered a useful DNA stain for short term UV exposure, its toxicity depends on the concentration, cell type, and exposure duration, emphasizing the need to evaluate its effects on oocyte and embryo viability [25]. In the present study, we evaluated the effects of different Hoechst 33342 concentrations and exposure durations on the development of zygotes with 2PN after centrifugation. The concentrations tested were 5 and 10 μg/mL, with exposure durations maintained within 30 min. These conditions are commonly used for chromosome staining during metaphase in somatic cell nuclear transfer procedures without impairing oocyte functionality [26].

Our results demonstrated that zygotes exposed to 5 μg/mL Hoechst 33342 for 30 min showed significantly higher blastocyst formation rates than the other treatment groups, highlighting the importance of optimizing staining conditions to preserve embryo viability. This finding is consistent with previous studies demonstrating that exposure of matured porcine oocytes to 5 μg/mL Hoechst 33342 for 30 min did not interfere with fertilization or subsequent blastocyst development [27]. Similarly, matured bovine oocytes exposed to the same concentration for 30 min followed by brief UV irradiation retained cytoplasmic viability [25]. However, previous studies have also reported that Hoechst 33342 staining combined with UV irradiation may impair embryonic development through mitochondrial dysfunction and reduced mitochondrial DNA copy number in early embryos [28]. Furthermore, increasing Hoechst 33342 concentrations to 7.5 and 10 μg/mL negatively affected embryo development in mice [29], which is consistent with the present findings showing reduced blastocyst development at 10 μg/mL Hoechst 33342.

In the present study, embryo quality was evaluated primarily based on blastocyst formation rates. However, additional developmental parameters, including total cell number, inner cell mass/trophectoderm (ICM/TE) ratio, apoptosis, and developmental kinetics, were not evaluated, which represents a limitation of this study. Future studies should incorporate these parameters to provide a more comprehensive assessment of embryo quality after live pronuclear staining.

### Effects of 2PN selection on genome editing efficiency and mosaicism

Most mutants generated from electroporated zygotes exhibit genetic mosaicism, in which individual cells carry different insertion or deletion (indel) patterns, suggesting that genome editing occurs after the first genome replication event [7, 30, 31]. This problem may be exacerbated when pronuclear status is not verified before electroporation. To synchronize genome editing events and reduce mosaicism, we selected embryos with 2PN at a defined time after fertilization. However, 2PN selection did not affect embryo development or improve the biallelic mutation rate and mutation efficiency in blastocysts after *TP53* gene targeting.

According to Sousa and Tesarik [32], approximately 5 h after insemination, the second polar body is extruded and the pronuclei enter the G1 phase. DNA replication subsequently begins during the S-phase and progresses through the G2 phase until chromosome condensation occurs during the M-phase, with pronuclear breakdown occurring during G2 before syngamy. In mice, DNA replication in one-cell embryos is generally asynchronous between maternal and paternal pronuclei, and only approximately 20% of embryos exhibit synchronized replication patterns. Replication usually begins earlier in the female pronucleus, whereas the male pronucleus either replicates later or follows a distinct replication pattern [10, 11]. Similarly, another study reported that the oocyte cytoplasm supports asynchronous initiation of DNA synthesis between the two pronuclei, with a time difference of at least 2 h [33]. In pigs, *in vitro* matured oocytes also exhibit asynchronous pronuclear formation, resulting in delayed first cleavage compared with *in vivo* matured oocytes [12]. This asynchrony may partly explain the reduced developmental competence observed under *in vitro* conditions, although the precise mechanisms remain unclear [34].

These observations suggest that selecting embryos with 2PN does not necessarily ensure synchronized DNA replication events between maternal and paternal pronuclei, which may explain why genome editing efficiency was not improved in the present study. Therefore, although pronuclear selection confirms normal fertilization, it may not adequately control the biological timing required for synchronized CRISPR/Cas9 activity before DNA replication.

### Biological implications and future perspectives

To our knowledge, this study is among the first to evaluate whether selecting zygotes with 2PN can synchronize genome editing before the first DNA replication event in electroporated porcine embryos. Despite successful selection of fertilized zygotes, biallelic mutation rates were not improved. These findings suggest that pronuclear asynchrony between maternal and paternal genomes may represent a biological barrier to achieving uniform genome editing in porcine embryos.

To directly test this hypothesis, future studies should incorporate DNA synthesis-labeling approaches, such as BrdU or EdU incorporation assays, to precisely determine the timing of S-phase progression within individual pronuclei. Such analyses would provide direct evidence linking pronuclear replication dynamics with genome editing outcomes. Furthermore, approaches involving replication control, pronuclear-specific delivery systems, and real-time monitoring of S-phase progression may provide promising strategies for improving genome editing precision in livestock embryos.

Comparison with a previous study using the same *TP53*-targeting gRNA demonstrated that the biallelic mutation rate in porcine blastocysts was approximately 60.0% [35], whereas the present study achieved rates of 81.8% and 78.6% in the non-selected and 2PN-selected groups, respectively. These findings suggest that the baseline genome editing efficiency in the present system was already relatively high, which may have limited the observable benefits of 2PN selection. However, detailed mosaicism patterns, including the number of indel variants and their distribution among embryonic cell lineages, were not evaluated in the present study. Future investigations should incorporate single-cell or lineage-specific analyses to better characterize mosaicism patterns after electroporation-mediated genome editing in porcine embryos. 

## CONCLUSION

 The present study demonstrated that the proportion of porcine zygotes with 2PN gradually increased after IVF and reached the highest level at 20 h after fertilization. Among the evaluated staining conditions, treatment with 5 μg/mL Hoechst 33342 for 30 min resulted in the highest blastocyst formation rate, indicating that this condition was suitable for live pronuclear visualization while maintaining embryo viability. However, selection of zygotes with 2PN before electroporation did not improve embryo development, biallelic mutation rates, or overall genome editing efficiency compared with non-selected embryos. These findings suggest that confirmation of normal fertilization based solely on 2PN visualization is insufficient to reduce mosaicism or enhance CRISPR/Cas9-mediated genome editing in porcine embryos.

The practical implication of this study is that optimization of pronuclear observation timing and Hoechst 33342 staining conditions can support reliable live embryo selection without substantial developmental toxicity. Nevertheless, routine application of 2PN selection before electroporation may have limited practical value because only a proportion of embryos exhibit visible 2PN at the optimal observation time, and the additional centrifugation and fluorescence-staining procedures increase procedural complexity without improving editing outcomes.

A major strength of this study was the integration of dynamic pronuclear assessment, optimized live-cell staining conditions, and electroporation-based genome editing within a single experimental workflow. In addition, this study provides important biological insight into the potential role of asynchronous pronuclear DNA replication as a limiting factor contributing to persistent mosaicism in porcine embryos. However, several limitations should be acknowledged. Detailed embryo quality parameters, including total cell number, inner cell mass/ trophic-toderm ratio, apoptosis, and developmental kinetics, were not evaluated. Furthermore, detailed mosaicism patterns and lineage-specific indel distributions were not analyzed, and off-target effects were not assessed.

Future studies should investigate strategies to synchronize genome editing with pronuclear DNA replication by incorporating approaches such as BrdU or EdU incorporation assays, replication control systems, and real-time monitoring of S-phase progression. In addition, advanced genome editing technologies, including base editing and pronuclear-specific delivery systems, may help reduce mosaicism and improve editing precision in porcine embryos.

Overall, the present findings indicate that although 2PN selection is safe for embryo development, it alone is insufficient to improve genome editing efficiency because of the biological asynchrony between maternal and paternal pronuclei. Further optimization of replication timing and genome editing synchronization strategies will be necessary to achieve more uniform and efficient genome editing in porcine embryos.

## DATA AVAILABILITY

All generated data are included in the manuscript.

## AUTHORS’ CONTRIBUTIONS

KT: Conceptualization, methodology, investigation, data curation, formal analysis, and writing – original draft. TO: Conceptualization, study design, supervision, validation, and manuscript reviewing and editing. MW, OSW, TT, and KC: Supervision, validation, and manuscript reviewing and editing. YN, ZN, MH, and FT: Experimental assistance, data collection, formal analysis, and statistical analysis. MN: Project administration, coordination of experiments, supervision, and manuscript reviewing and editing. All authors contributed to the work described in this manuscript and have read and approved the final version of the manuscript.
